# Genome-Wide Association Study of Male Sexual Orientation

**DOI:** 10.1038/s41598-017-15736-4

**Published:** 2017-12-07

**Authors:** Alan R. Sanders, Gary W. Beecham, Shengru Guo, Khytam Dawood, Gerulf Rieger, Judith A. Badner, Elliot S. Gershon, Ritesha S. Krishnappa, Alana B. Kolundzija, Jubao Duan, MGS Collaboration, Jianxin Shi, Douglas F. Levinson, Bryan J. Mowry, Ann Olincy, Farooq Amin, C. Robert Cloninger, Dragan M. Svrakic, Jeremy M. Silverman, Nancy G. Buccola, William F. Byerley, Donald W. Black, Robert Freedman, Pablo V. Gejman, J. Michael Bailey, Eden R. Martin

**Affiliations:** 1 Department of Psychiatry and Behavioral Sciences, NorthShore University HealthSystem Research Institute, Evanston, Illinois 60201 United States of America; 2 Department of Psychiatry and Behavioral Neuroscience, University of Chicago, Chicago, Illinois 60637 United States of America; 3 Department of Human Genetics, University of Miami, Miami, Florida 33136 United States of America; 4 Department of Psychology, Pennsylvania State University, University Park, Pennsylvania, 16802 United States of America; 5 Department of Psychology, University of Essex, Colchester, England CO4 3SQ United Kingdom; 6 Department of Psychiatry, Rush University Medical Center, Chicago, Illinois 60612 United States of America; 7 Department of Psychiatry, Icahn School of Medicine at Mount Sinai, Elmhurst, New York, 11373 United States of America; 8 Department of Sociomedical Sciences, Mailman School of Public Health, Columbia University, New York, New York, 10027 United States of America; 9 Department of Psychology, Northwestern University, Evanston, Illinois 60208 United States of America; 10 Division of Cancer Epidemiology and Genetics, National Cancer Institute, Bethesda, Maryland, 20892 United States of America; 11 Department of Psychiatry and Behavioral Sciences, Stanford University, Stanford, California, 94305 United States of America; 12 Queensland Centre for Mental Health Research, Brisbane and Queensland Brain Institute, The University of Queensland, Brisbane, Queensland, 4072 Australia; 13 Department of Psychiatry, University of Colorado Denver, Aurora, Colorado 80045 United States of America; 14 Department of Psychiatry and Behavioral Sciences, Atlanta Veterans Affairs Medical Center, and Emory University, Atlanta, Georgia, 30329 United States of America; 15 Department of Psychiatry, Washington University, St. Louis, Missouri 63110 United States of America; 16 Department of Psychiatry, Mount Sinai School of Medicine, New York, New York 10029 United States of America; 17 School of Nursing, Louisiana State University Health Sciences Center, New Orleans, Louisiana 70112 United States of America; 18 Department of Psychiatry, University of California at San Francisco, San Francisco, California, 94143 United States of America; 19 Mental Health Clinical Research Center, and Department of Psychiatry, University of Iowa Carver College of Medicine, Iowa City, Iowa 52242 United States of America

## Abstract

Family and twin studies suggest that genes play a role in male sexual orientation. We conducted a genome-wide association study (GWAS) of male sexual orientation on a primarily European ancestry sample of 1,077 homosexual men and 1,231 heterosexual men using Affymetrix single nucleotide polymorphism (SNP) arrays. We identified several SNPs with *p* < 10^−5^, including regions of multiple supporting SNPs on chromosomes 13 (minimum *p* = 7.5 × 10^−7^) and 14 (*p* = 4.7 × 10^−7^). The genes nearest to these peaks have functions plausibly relevant to the development of sexual orientation. On chromosome 13, *SLITRK6* is a neurodevelopmental gene mostly expressed in the diencephalon, which contains a region previously reported as differing in size in men by sexual orientation. On chromosome 14, *TSHR* genetic variants in intron 1 could conceivably help explain past findings relating familial atypical thyroid function and male homosexuality. Furthermore, skewed X chromosome inactivation has been found in the thyroid condition, Graves’ disease, as well as in mothers of homosexual men. On pericentromeric chromosome 8 within our previously reported linkage peak, we found support (*p* = 4.1 × 10^−3^) for a SNP association previously reported (rs77013977, *p* = 7.1 × 10^−8^), with the combined analysis yielding *p* = 6.7 × 10^−9^, i.e., a genome-wide significant association.

## Introduction

While the usual combination of sex chromosomes (XX or XY) predicts sexual orientation and behavior for the vast majority of humans (as heterosexual), variation exists: a stable minority of men (3~4%) are homosexual^1^ and male sexual orientation appears to be bimodally distributed with most men rating themselves as predominantly heterosexual (Kinsey scale 0–1) or homosexual (Kinsey scale 5–6)^1–5^. Male sexual orientation is moderately heritable (30~40%), but is multifactorial, with evidence of multiple genetic and environmental contributions, via family studies^6–11^, twin studies^4,12–16^, and segregation analyses^8,10,11,17^. Although focused (i.e., chromosome X) and genome-wide linkage studies (GWLS) of affected (i.e., concordant) sibling pairs have been applied to the trait^8,18–22^, these have been in relatively small samples prior to our own GWLS^23^, which showed genome-wide significant linkage to the pericentromeric region of chromosome 8 and strong support for linkage to the previously reported Xq28 region. Although genetic variant(s) contributing to development of male sexual orientation have been mapped to pericentromeric chromosome 8 and chromosome Xq28, the linkage peaks are large and specific trait genes have not been identified.

Genetic association studies for male sexual orientation have been sparse, small, and used either a convenience sample with proxy markers (e.g., blood type) or usually a candidate gene approach, and have yielded negative findings (strongest finding being a nominal *p* = 0.03)^24–27^. No genome-wide association studies (GWAS) for male sexual orientation have been heretofore published in the peer-reviewed literature. One meeting report (2012 ASHG) from the company 23andMe analyzed male sexual orientation (N = 13,733 European ancestry men) as a continuous variable^28^ and identified its strongest association (*p* = 7.1 × 10^−8^, direction unstated) in rs77013977^29^, an intronic SNP in *NKAIN3* (*Na*+/*K*+ *transporting ATPase interacting 3*), which lies in a multipoint linkage peak on the pericentromeric region of chromosome 8 identified by our lab^23^. To extend our gene mapping efforts for the trait, we report here the results from analyzing 1,109 homosexual and 1,231 heterosexual primarily European ancestry men in the first published GWAS on the trait.

## Results/Discussion

**Figure 1:**
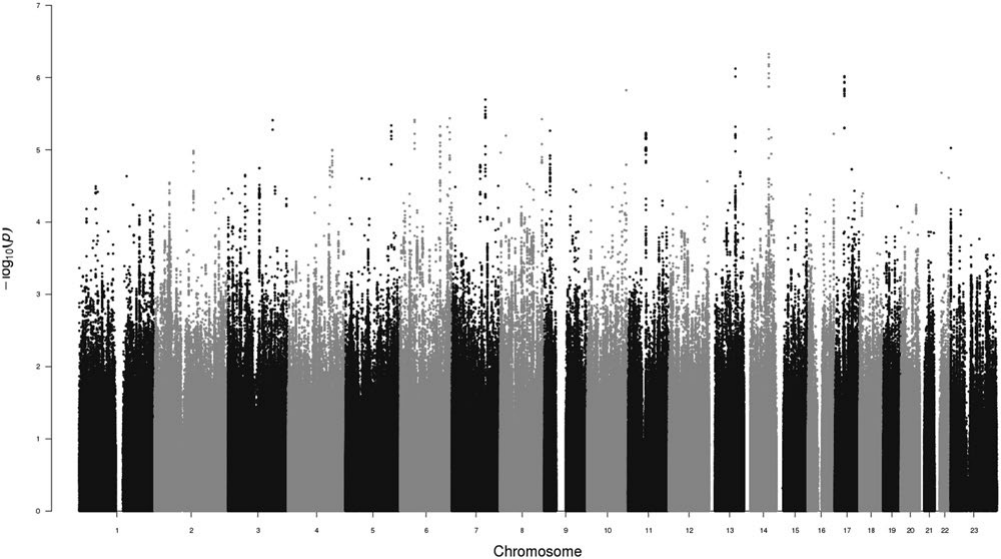
Manhattan Plot of GWAS for Male Sexual Orientation. Plot of negative log_10_ of the *p*-values for the single SNP association analysis of 1,077 homosexual and 1,231 heterosexual men, ordered along the x-axis for each chromosome by chromosomal position.

We detected several promising regions of multiple SNPs in the 10^−5^–10^−7^ *p*-value range, as seen in the Manhattan plot (Fig. 1), though no SNP reached genome-wide significance (5 × 10^−8^). The most prominent of these regions were on chromosomes 13 (minimum *p* = 7.5 × 10^−7^, rs9547443) and 14 (*p* = 4.7 × 10^−7^, rs1035144), where some SNPs had 10^−7^ < *p* < 10^−6^ (each region with 9 to 10 SNPs with *p* < 10^−5^, Table S1). There are a number of genes of relevance to the trait in and around these regions, which we describe below. We further note that the most significant SNP (rs77013977, *p* = 7.1 × 10^−8^) in the 23andMe male GWAS^29^ was nominally associated (*p* = 4.1 × 10^−3^) in our own GWAS. We used a meta-analytic statistic that did not need direction of effect, Fisher’s combined probability test^30^, which yielded *p* = 6.7 × 10^−9^ for this SNP, which is the first reported genome-wide significant association for the trait. As previously noted^29^, rs77013977 is an intronic SNP in *NKAIN3*, which is one of a family of four proteins (NKAIN1–4) suggested to be critical for neuronal function^31^.

The strongest associated region on chromosome 13 (rs9547443, *p* = 7.5 × 10^−7^) was located between *SLITRK6* (*SLIT and NTRK like family member 6*, ~60 kb centromeric to region) and *SLITRK5* (~1.8 Mb telomeric), with *SLITRK1* located ~2.0 Mb centromeric. Members of the SLITRK protein family are brain-expressed neuronal transmembrane proteins that regulate neuronal outgrowth, survival, and synapse formation; SLITRKs have significant homology to the secreted axonal growth-controlling SLIT family of proteins and also homology to the neurotrophic tyrosine kinase receptor (NTRK) family^32–34^. *SLITRK6* is expressed especially in the diencephalon (which contains a region previously reported as differing in size in men by sexual orientation^35^), and *SLITRK1* and *SLITRK5* have their highest expression in the cerebral cortex^32–34^. Gene families, such as the SLITRK family that are important for neurodevelopment and are implicated as candidate genes for various neuropsychiatric phenotypes^34^, are also of potential relevance to behavioral phenotypes such as sexual orientation.

On chromosome 14, *TSHR* (*thyroid stimulating hormone receptor*) spans the region around our most significant SNP (rs1035144, *p* = 4.7 × 10^−7^), and includes a cluster of SNPs with association *p* < 10^−5^ in intron 1. *TSHR* encodes a G protein-coupled transmembrane receptor for thyrothropin (thyroid stimulating hormone) and thyrostimulin, manifests some constitutive activity (i.e., ligand independent), and is a major controller of thyroid cell metabolism^36–38^. While the main tissue of interest and expression for *TSHR* is the thyroid gland, *TSHR* is expressed in other tissues including brain especially in neuron-rich areas (e.g., hippocampus)^39^. *TSHR* codes for the major autoantigen in the autoimmune hyperthyroidism of Graves’ disease, which is associated (*p* < 10^−20^ with OR’s 1.4~1.5) with intron 1 polymorphisms^40–48^. A recent population-based study found that 5,351 same-sex married men among the assayed population of 2,252,751 Danish men had an elevated rate ratio of Graves’ disease (RR = 1.88; 95% CI = 1.08–3.01), a finding which held when excluding men with HIV/AIDS^49^. The authors^49^ speculate on the possibility that a genetic (or other prenatal) factor might tie together this increased risk for a type of hyperthyroidism (Graves’ disease) with separate observations of lower body weight for homosexual versus heterosexual men (independent of diet or exercise)^50–52^. Females with Graves’ disease have been reported to manifest biased X chromosome inactivation^53–55^, and skewed X chromosome inactivation has also been reported in mothers of homosexual men compared to age-matched mothers of heterosexual men^56^. Furthermore, a recent retrospective chart review of 790 adolescents (8 to 17 years) previously admitted to a child psychiatry service found 15 mothers with a history of thyroid dysfunction during pregnancy, 16 adolescents with a history of same-sex attraction and/or gender nonconformity, and 12 overlapping mother-offspring pairs with both (*p* < 0.0001), suggestive of a possible relationship^57^. Thus converging findings, including suggestive evidence from the current study, point to a possible connection between thyroid function and sexual orientation in men.

The main limitations of the current study include an exclusive focus on males, sampling primarily from one ancestral group (European), combination of two datasets, and most notably the modest sample size for a GWAS on a trait with complex genetics. Additional and larger sample sizes would be required to assess which loci might breach genome-wide significance for association in a single study, and to increase the number of such loci (as typically is the case with phenotypes with manifesting complex genetics^58,59^). Nevertheless, our study provides support for the top finding from a previous meeting report of a GWAS on the trait^29^, reaching genome-wide significance for the combined analysis of rs77013977 (*p* = 6.7 × 10^−9^) on pericentromeric chromosome 8. In addition, the current study’s top two association peaks (*p* < 10^−5^; Fig. 1) provide interesting and perhaps trait-relevant examples of their closest genes on chromosomes 13 (*SLITRK6*) and 14 (*TSHR*), though these potential connections are best characterized as speculative. The continued genetic study of male sexual orientation should help open a gateway to other studies focusing on genetic and environmental mechanisms of sexual orientation and development. Detectable genetic variants predisposing to homosexuality would have alternative alleles, which would necessarily predispose to heterosexuality, thus contributing to understanding of both typical heterosexual and minority homosexual orientations.

## Methods

### Study Sample

We obtained institutional review board approval from NorthShore University HealthSystem, and after a study description all enrolled subjects gave informed consent. All methods were performed in accordance with the relevant guidelines and regulations. Our GWAS analyzed sample of primarily European ancestry men included a total of 1,077 homosexual and 1,231 heterosexual men, comprised as follows. In our GWLS on 409 pairs of homosexual brothers in 384 multiplex families^23^, we classified men as homosexual based on both their self-reported sexual identity and sexual feelings (Kinsey 5–6). Isolated DNA samples were genotyped (Affymetrix 5.0 SNP array) at Vanderbilt Microarray Shared Resource. For this GWAS, after removing 12 families (two homosexual brothers each) for being ancestry outliers via principal components analysis (PCA), we included all remaining homosexual males (N = 769) and all heterosexual males (N = 33) from the GWLS dataset (372 families, each with two or more homosexual brothers, Supplementary Table S2). In addition, we genotyped (same Affymetrix 5.0 platform) additional males (same phenotypic definitions), with the following subjects being retained after quality control (QC, below): 221 homosexual males and 13 heterosexual males (from 227 partially completed linkage families, i.e., reported to contain two or more homosexual brothers, Supplementary Table S2), and 51 sporadic homosexual males (i.e., without homosexual brothers). The aforementioned subjects were collected as previously described^23^, largely from community festival venues. We also incorporated our Molecular Genetics of Schizophrenia (MGS) collaboration controls dataset (the male, European ancestry portion retained after QC: 36 homosexual and 1,185 heterosexual) that was genotyped with Affymetrix 6.0 at the Broad Institute, and was queried regarding sexual orientation (identity)^60,61^.

### Statistical analysis

As part of the QC design to help minimize errors due to platform-specific genotype calling differences, we genotyped 34 duplicate subjects on both platforms (Affymetrix 5.0 and 6.0), removing SNPs discrepant for any of the 34 inter-platform duplicates (Supplementary Table S3). Our further standard GWAS QC^62,63^ filters included removal of SNPs (minor allele frequency, MAF < 0.05; missingness ≥1%; Hardy-Weinberg equilibrium [HWE] deviation *p* < 10^−6^; Supplementary Table S4) and removal of samples (missingness > 5%; failing checks for duplications and relatedness; ancestry outliers via PCA; Supplementary Table S3, and also see Supplementary Figure S1 for plotting of PC1 and PC2), resulting in λ_1000_ = 1.029 (i.e., a low genomic inflation factor). For the X chromosome, we followed the same QC protocol, but estimated HWE only in females (mothers in multiplex families). The pseudoautosomal regions (PAR) were removed prior to analysis. We imputed to 1,000 Genomes^64^ using the IMPUTE2 software^65^ (removing SNPs with an information score < 0.6, MAF < 0.05) prior to performing GWAS analyses. The final QC’d SNP dataset contained a total of 5,642,880 retained SNPs (200,367 typed and 5,442,513 imputed). Association analysis was conducted using logistic regression with typed and imputed data in the R package, Genome-Wide Association analyses with Family (GWAF)^66^, with PC1 and PC2 as covariates. Regional association plots for the two regions highlighted in Supplementary Table S1 are displayed in Supplementary Figure S2.

### Data Availability

The MGS collaboration controls dataset has been previously deposited into the database of Genotypes and Phenotypes (dbGaP, dbgap.ncbi.nlm.nih.gov, phs000021 and phs000167). The GWLS dataset generation was previously described^23^. For all studied subjects, additional information is included within the accompanying supporting information (Supplementary Tables S1–S4 and Supplementary Figures S1-S2). The results from all data analyzed during this study are included or displayed in this published article (and its Supplementary Information files, such as the Manhattan plot and PCA plot), though only top individual SNP results for the two strongest regions are tabulated (Table S1) and regionally displayed (Figure S2). However, other individual SNP results may be made available from the corresponding author on reasonable request.

## Electronic supplementary material

Supplementary Information
