# Supplementary material for: Genome-Wide Association Study of Male Sexual Orientation
Source: Sci Rep. 2017 Dec 7;7:16950. doi: 10.1038/s41598-017-15736-4 (PMC5721098; doi:10.1038/s41598-017-15736-4)
Supplement: Supplementary file 1 — Supplementary Information [file 41598_2017_15736_MOESM1_ESM.pdf]

Genome-Wide Association Study of Male Sexual Orientation

Alan R. Sanders[1,2,*], Gary W. Beecham[3], Shengru Guo[3], Khytam Dawood[4], Gerulf Rieger[5], Judith A. Badner[6], Elliot S. Gershon[2], Ritesha S. Krishnappa[7], Alana B. Kolundzija[8], Jubao Duan[1,2], MGS Collaboration[+], Pablo V. Gejman[1,2], J. Michael Bailey[9], Eden R. Martin[3]

Supplementary Information

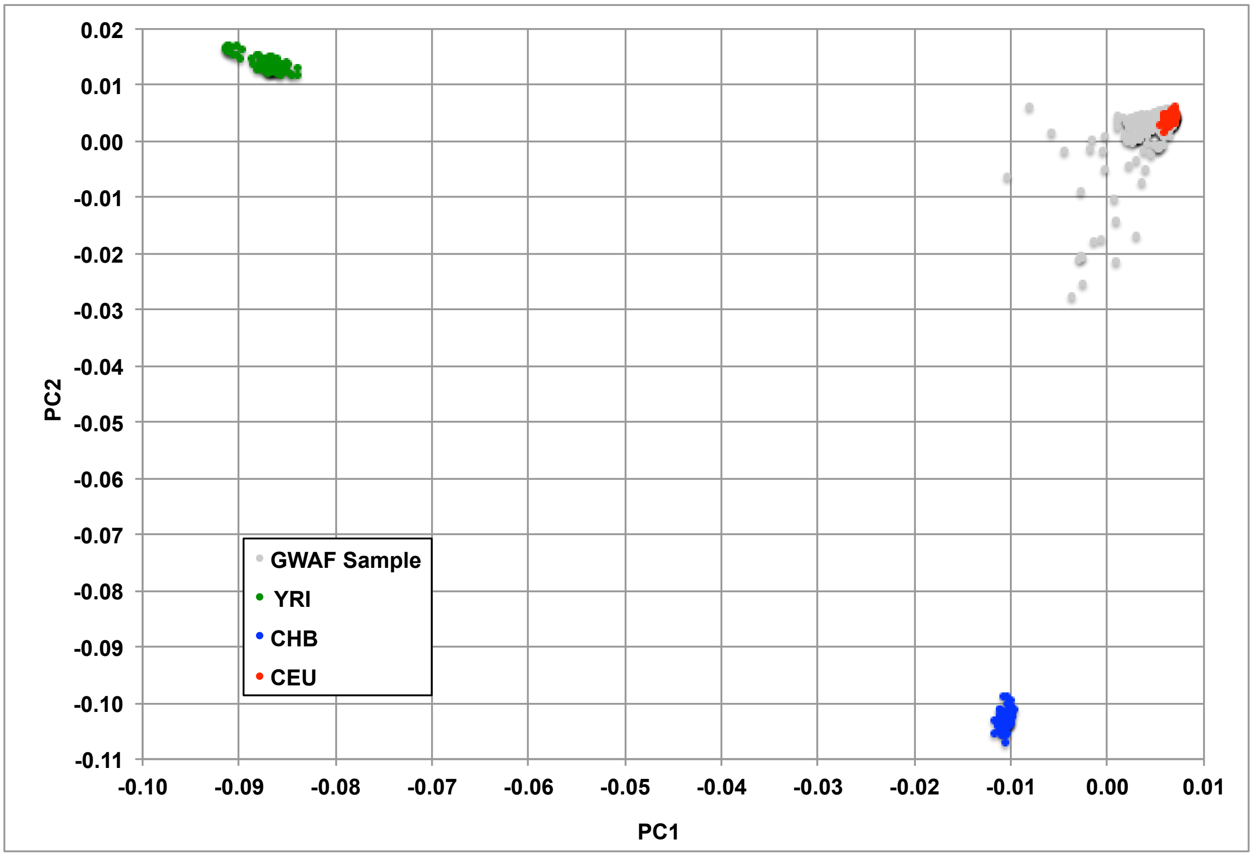

Supplementary Figure S1. PCA Plot of Top Two PC's of Study Samples and HapMap Reference Populations. Plot of PC1 and PC2 for one randomly chosen individual in each of 1,871 analyzed families along with 317 unrelated individuals from CEU, YRI and CHB HapMap reference populations [67].

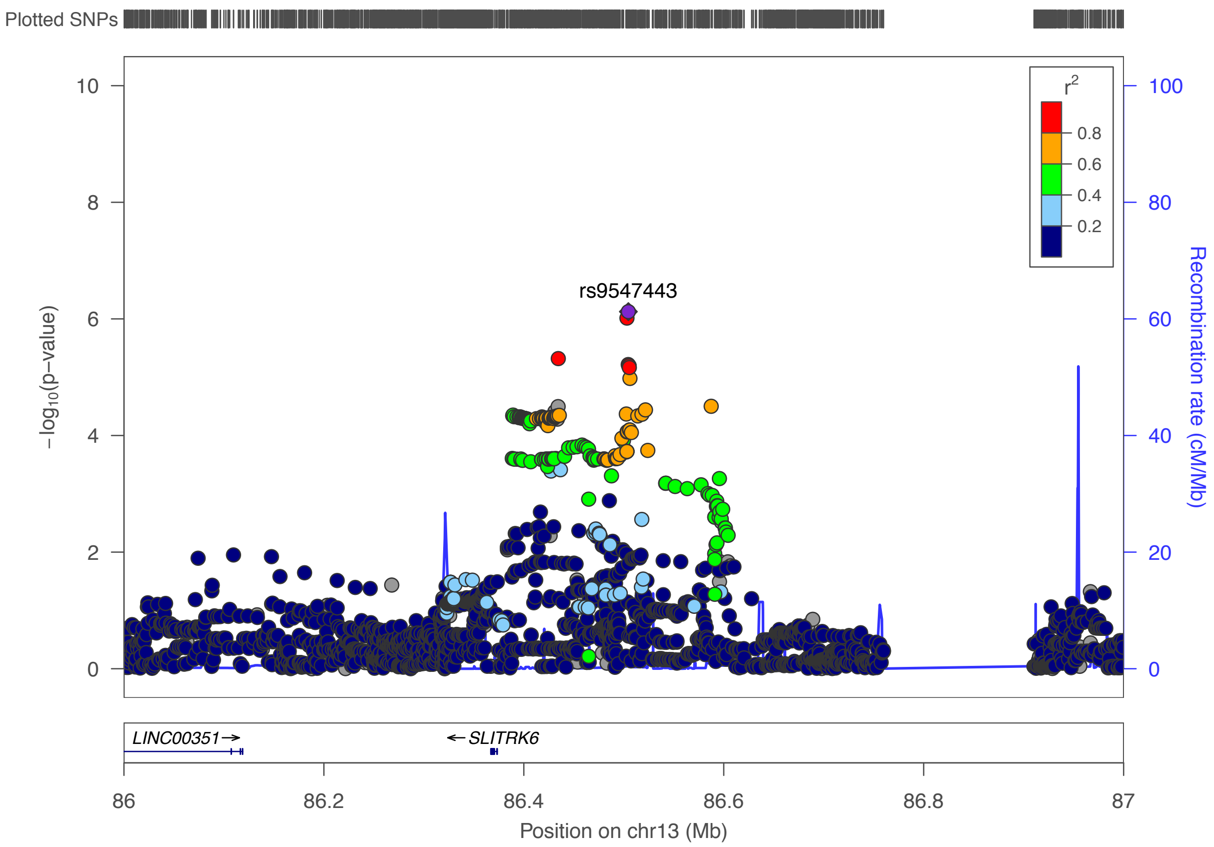

Plotted SNPs
rs9547443
r2
0.8
0.6
0.4
0.2
−log10(p−value)
Recombination rate (cM/Mb)
LINC00351→
← SLITRK6
Position on chr13 (Mb)

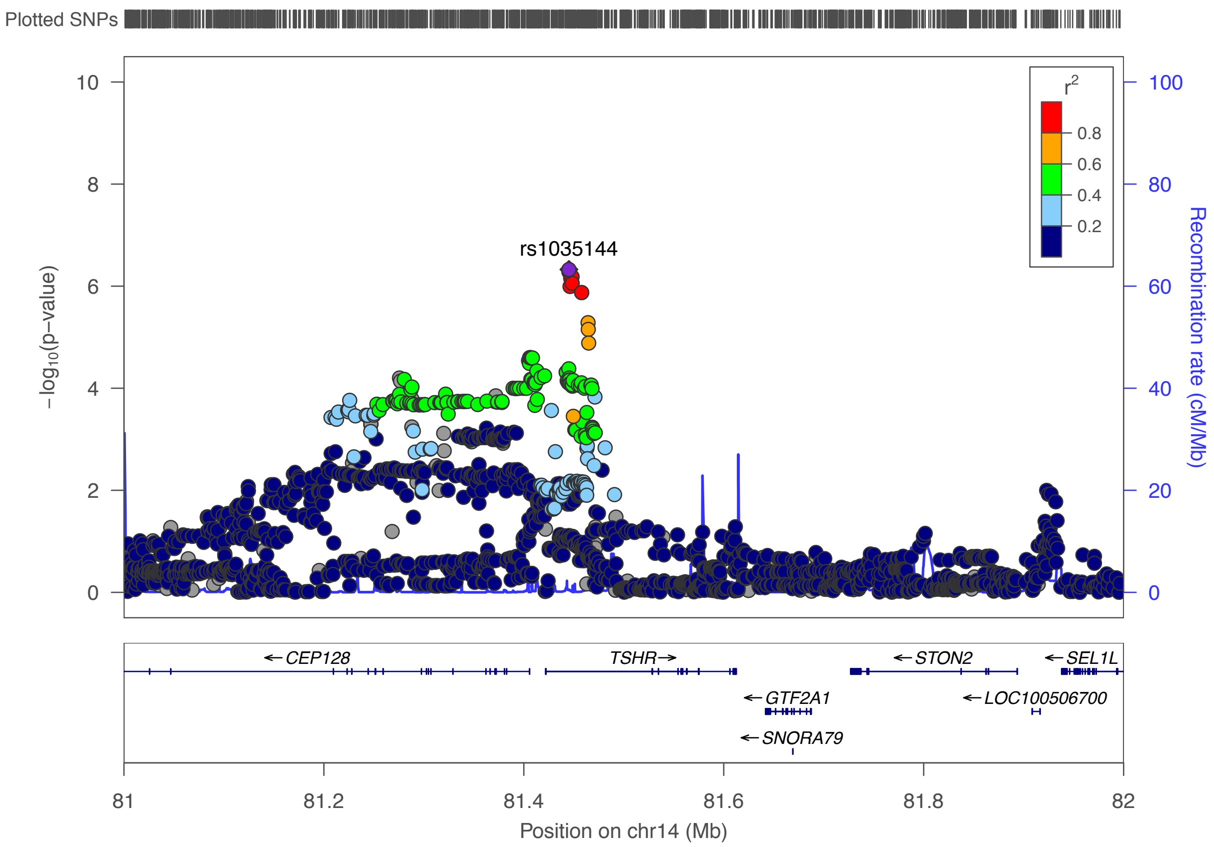

Supplementary Figure S2. Regional Association Plots of Regions in Supplementary Table S1. These plots indicate position (GRCh37/hg19), strength of association by -$\log_{10}$(p-value), and recombination rate (cM/Mb), with r^2 to the most associated SNP color coded as indicated. Density of assayed SNPs is indicated above, and nearby genes below the plot. The plotted regions are from 86-87 Mb on chromosome 13 (**A**) and from 81-82 Mb on chromosome 14 (**B**).

Chr	bp	SNP	EA	RA	EAF	info	beta	se	*p*-value
13	86,434,490	rs9547412	A	G	0.710	0.992	0.340	0.074	4.79E-06
13	86,503,179	rs4312206	G	A	0.720	0.980	0.370	0.076	9.68E-07
13	86,504,474	rs9531890	A	G	0.736	0.986	0.343	0.076	6.07E-06
13	86,504,577	rs9547443	C	T	0.719	0.988	0.371	0.075	7.51E-07
13	86,504,968	rs75843441	C	CTTG	0.734	0.990	0.342	0.076	6.39E-06
13	86,505,172	rs9547444	T	C	0.734	0.989	0.342	0.076	6.39E-06
13	86,505,211	rs9547445	A	G	0.734	0.989	0.342	0.076	6.39E-06
13	86,505,374	rs9547446	G	A	0.734	0.989	0.342	0.076	6.39E-06
13	86,505,542	rs9547447	G	A	0.733	0.986	0.341	0.076	6.83E-06
14	81,445,108	rs4411444	A	G	0.510	0.988	-0.327	0.065	5.24E-07
14	81,445,121	rs1035144	C	T	0.509	0.988	-0.328	0.065	4.71E-07
14	81,446,242	rs8003515	G	C	0.515	0.979	-0.320	0.065	1.01E-06
14	81,446,908	rs2300516	A	G	0.512	0.989	-0.322	0.065	6.96E-07
14	81,448,282	rs3783950	C	G	0.512	0.989	-0.323	0.065	6.55E-07
14	81,448,382	rs3783949	T	G	0.525	0.990	-0.318	0.065	8.76E-07
14	81,457,788	rs2371463	G	A	0.525	0.973	-0.316	0.065	1.34E-06
14	81,457,940	rs724170	G	A	0.525	0.973	-0.316	0.065	1.33E-06
14	81,464,279	rs12323356	A	G	0.551	0.946	-0.305	0.067	5.19E-06
14	81,464,486	rs4903962	A	G	0.551	0.946	-0.301	0.067	7.03E-06

Supplementary Table S1: Regions on chromosomes 13 and 14 with SNPs associated at $p<10^{-5}$. Note: Base pair location per Human Feb. 2009 (GRCh37/hg19); EA, RA, and EAF refer to the effect allele, reference allele, and effect-allele frequency, respectively.

Number of homosexual brothers/family	Family count	Number of homosexual brothers	Number of heterosexual brothers	Number of total brothers
0	6	0	6	6
1	221	221	7	228
2	349	698	32	730
3	21	63	1	64
4	2	8	0	8
Totals	599	990	46	1,036

Supplementary Table S2: SNP genotyped individuals from linkage families analyzed for GWAS. Note: These counts reflect 802 typed brothers from 372 multiplex families previously analyzed for linkage [23], and 234 typed brothers from 227 multiplex families not analyzed for linkage (since not enough brothers were collected).

Platform	Affymetrix 5.0	Affymetrix 6.0	Totals
Original Sample N	1,180	1,226	2,406
Dropped for missingness >=5%	2	4	6
Dropped from duplication checks	5	1	6
Dropped from relatedness checks	9	11	20
Dropped for ancestry outliers	32	0	32
Remaining Sample N	1,132	1,210	2,342

Supplementary Table S3: Sample QC. Note: One member of each the 34 inter-platform (Affymetrix 5 & Affymetrix 6) duplicate pairs was removed, leaving 2,308 analyzed samples.

<table>
<tr><th>Platform</th><th>Affymetrix 5.0</th><th>Affymetrix 6.0</th></tr>
<tr><td>Original SNPs (N)</td><td>440,793</td><td>436,218</td></tr>
<tr><td>Dropped for missingness >=5%</td><td>32,481</td><td>30,045</td></tr>
<tr><td>Dropped HWE deviation p<0.000001</td><td>11</td><td>861</td></tr>
<tr><td>Dropped due to non-overlapping SNP (i.e., missing from other platform)</td><td>24,242</td><td>21,253</td></tr>
<tr><td>Dropped due to discrepancy for any of the 34 inter-platform duplicates</td><td>22,189</td><td>22,189</td></tr>
<tr><td>Remaining merged (Affy 5 & Affy 6) SNP N</td><td>361,870</td><td>361,870</td></tr>
<tr><td>Merged SNP N</td><td colspan="2">361,870</td></tr>
<tr><td>Dropped due to not mapping to Chr1-Chr23</td><td colspan="2">478</td></tr>
<tr><td>Dropped prior to imputation for unresolvable strand issues, e.g., A/T and C/G SNPs with similar allele frequencies</td><td colspan="2">59</td></tr>
<tr><td>Dropped prior to imputation for mismatching typed alleles to reference SNPs even after addressing strand inconsistencies</td><td colspan="2">252</td></tr>
<tr><td>After imputation, the total remaining typed SNPs</td><td colspan="2">361,081</td></tr>
</table>

Supplementary Table S4: SNP QC. Note: After imputation, typed SNPs were further filtered to only retain for analysis those SNPs with MAF >=5% and missingness <1%, resulting in 200,367 typed SNPs for analysis.
